# Larval flushing alters malaria endemicity patterns in regions with similar habitat abundance

**DOI:** 10.1016/j.crpvbd.2022.100080

**Published:** 2022-02-08

**Authors:** Vardayani Ratti, Jonathan W. Chipman, Dorothy I. Wallace

**Affiliations:** a Department of Mathematics and Statistics, California State University Chico, CA, USA; b Department of Geography, Dartmouth College, Hanover, NH, USA; c Department of Mathematics, Dartmouth College, Hanover, NH, USA

**Keywords:** *Anopheles gambiae*, Malaria, Flushing, Mathematical model, *Plasmodium falciparum*

## Abstract

A model of *Anopheles gambiae* populations dynamics coupled with *Plasmodium falciparum* transmission dynamics is extended to include mechanisms of larval flushing which are known to occur. Flushing dynamics are modeled using a simulation that incorporates seasonal, autocorrelated, and random components based on 30 years of rainfall data for the Kakamega District of the western Kenya highlands. The model demonstrates that flushing phenomena can account for differences between regions with the same annual larval habitat pattern, changing the World Health Organization endemicity classification from either hyperendemic or holoendemic to hypoendemic disease patterns. Mesoendemic patterns of infection occur at the boundary of the holoendemic to hypoendemic transition. For some levels of flushing the entomological inoculation rate drops to an insignificant amount and disease disappears, while the annual indoor resting density remains well above zero. In these scenarios, the disease is hypoendemic, yet the model shows that outbreaks can occur when disease is introduced at particular time points.

## Highlights

• A mathematical model demonstrates the effect of larval flushing rate and frequency on patterns of malaria endemicity.
• Flushing events are driven by a Monte Carlo simulation of daily rainfall.
• Four known endemic patterns (hypo-, hyper-, meso- and holoendemic) are produced by the model, and no others.
• Simulated flushing produces mesoendemic disease patterns at steady state, not seen in the absence of flushing.
• Hyperendemic transmission patterns may be reduced to hypoendemic with sufficient flushing.

## Graphical abstract

**Figure:**
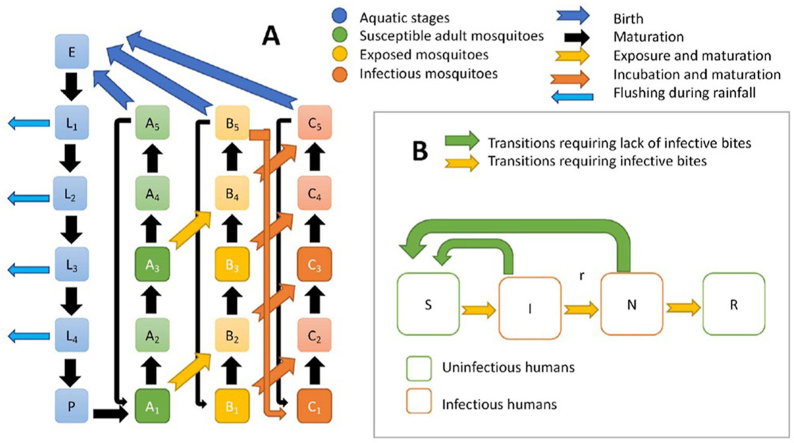

## 1 Introduction

Malaria is endemic in many countries, especially in sub-Saharan Africa, in spite of reported declines (Thellier et al., 2020). Most death is due to the species *Plasmodium falciparum*, carried by the *Anopheles gambiae* mosquito vector (Thellier et al., 2020). Four transmission classes have been proposed in the past by the WHO and commonly used by malaria researchers. Hypoendemic transmission is characterized by 0–10% prevalence, little transmission, and very low entomological inoculation rate (EIR, number of infectious bites per human per day or year) (Baird et al., 2002). Mesoendemic transmission is characterized by 11–50% prevalence, fluctuating transmission pattern and less than 10 EIR per day (Bruce-Chwatt et al., 1973; Baird et al., 2002; Doolan et al., 2009). In hyperendemic transmission both the EIR and prevalence are seasonal, with over 50% prevalence, high prevalence in adults, and low levels of immunity (Last et al., 2001; Baird et al., 2002). Holoendemic transmission is year-round with consistently high EIR, prevalence over 75%, low transmission in adults, and high immunity levels in adults (Last et al., 2001; Baird et al., 2002; Doolan et al., 2009).

In a prior modeling study, we were able to reproduce three out of these four patterns of transmission, along with expected immunity levels, simply by varying the extent to which larval habitat depends on rainfall (Ratti & Wallace, 2020). A detailed vector population model was based on the temperature and rainfall pattern in the western Kenya highlands. The mosquito population model was coupled with a transmission model to produce prevalence and immunity patterns in the simulated human population. The model produced holoendemic disease patterns when habitat was high, hyperendemic disease patterns when habitat was moderate and highly seasonal, and hypoendemic disease patterns when habitat was low.

Rainfall is correlated with larval vector habitat and yet in the western Kenya highlands nearby towns may have malaria transmission patterns that are different enough to fall into different WHO classes (Koenraadt et al., 2004; Githeko et al., 2006). This study investigates the extent to which this variability may be attributed to the flushing of larvae during heavy rains, which depends on factors independent of rainfall, such as topography and land use. Field observations from the western Kenya highlands indicate that the abundance of *An. gambiae* larvae may either be enhanced or diminished by increased rainfall (Imbahale et al., 2011). The reason for the increase is clear: more aquatic habitat is available. Diminished numbers after rainfall are often attributed to the flushing of larvae from these habitats by heavy rain. Direct measurements of flushing are usually made using full artificial containers which are sometimes left outdoors as rainfall is monitored and sometimes flooded with artificial rainfall (Paaijmans et al., 2007; Dieng et al., 2012). Larvae are placed in these containers and water added. Those that have been flushed over the sides are collected and counted. Early instars are flushed at a higher rate than late ones.

In these experiments, even a small amount of additional water causes flushing, as larvae that stray over the side of the container can never make their way back into it. In natural environments, the effect would be less drastic and would be likely to depend on the slope of the surrounding area. Mosquito larvae can survive for a while on wet land and can even crawl back to their puddle, although it is likely that this would be more difficult on a slope (Koenraadt et al., 2003; Fritz et al., 2012). Larvae can also dive, which may assist them in evading surface currents (Phelan & Roitberg, 2013).

When a flushing event occurs, it will remove a fraction of the larvae. This rate of removal depends on the intensity and duration of precipitation and on landscape characteristics including soil moisture and other soil properties, land cover, slope and slope length, surface roughness and structures, and other characteristics of the upslope drainage area. In general, for a given unit of precipitation, runoff will be highest and flushing most effective when (i) antecedent soil moisture is high (Todini, 1996; Olson et al., 2021), (ii) slopes are steep and slope lengths are long (Neal, 1938; Liu et al., 2000; Jain et al., 2004), (iii) surface roughness is low, and runoff control structures are absent (Battany & Grismer, 2000; Taye et al., 2013), and (iv) vegetation cover is low and is composed of pasture or cropland rather than mixed vegetation or forest (Peng & Wang, 2012; Taye et al., 2013; Guzha et al., 2018). However, the processes involved can be complex, e.g. soil surface processes can outweigh the effect of slope length (Bryan & Poesen, 1989). When gridded data or estimates of these properties are available, spatial models can be used to predict the spatiotemporal distribution of runoff from a precipitation event (Jain et al., 2004). Following precipitation and runoff, the flushing process will depend on the characteristics of individual habitats (e.g. depth), and of the larvae themselves, with lighter, earlier instars flushed at a higher rate than older, heavier instars (Paaijmans et al., 2007).

We extend the model developed in Ratti & Wallace (2020), which includes both the temperature- and habitat-dependent vector dynamics of *An. gambiae* and the transmission of *P. falciparum*. That model includes two types of human immunity which, coupled with temperature and habitat driven population dynamics, produces three of the four WHO endemic classifications for malaria. It was not observed to produce any endemicity patterns other than those observed; however, it did not produce mesoendemic disease. The revised model used in the present study includes linear terms describing death due to flushing events for all larval stages. Discrete flushing events are included when rainfall exceeds a chosen threshold. We created a Monte Carlo rainfall simulation after Benth et al. (2007), comprising deterministic, recursive, and random components with parameters derived from 30 years of rainfall data for a region in Kenya in the Kakamega District (Benth et al., 2007; Sreekanth et al., 2020). This simulation is used to select days when rainfall exceeds a given threshold in order to reduce larval populations at a given rate. Rainfall scenarios which produce hyperendemic and holoendemic transmission patterns with no flushing effects, taken from Ratti & Wallace (2020) are extended to include flushing. Numerical experiments that vary the threshold rainfall required to flush larvae, and the rate at which those larvae are removed, are designed to establish conditions under which the same rainfall pattern may lead to malaria transmission patterns in multiple WHO classifications.

## 2 Materials and methods

**Fig. 1:**
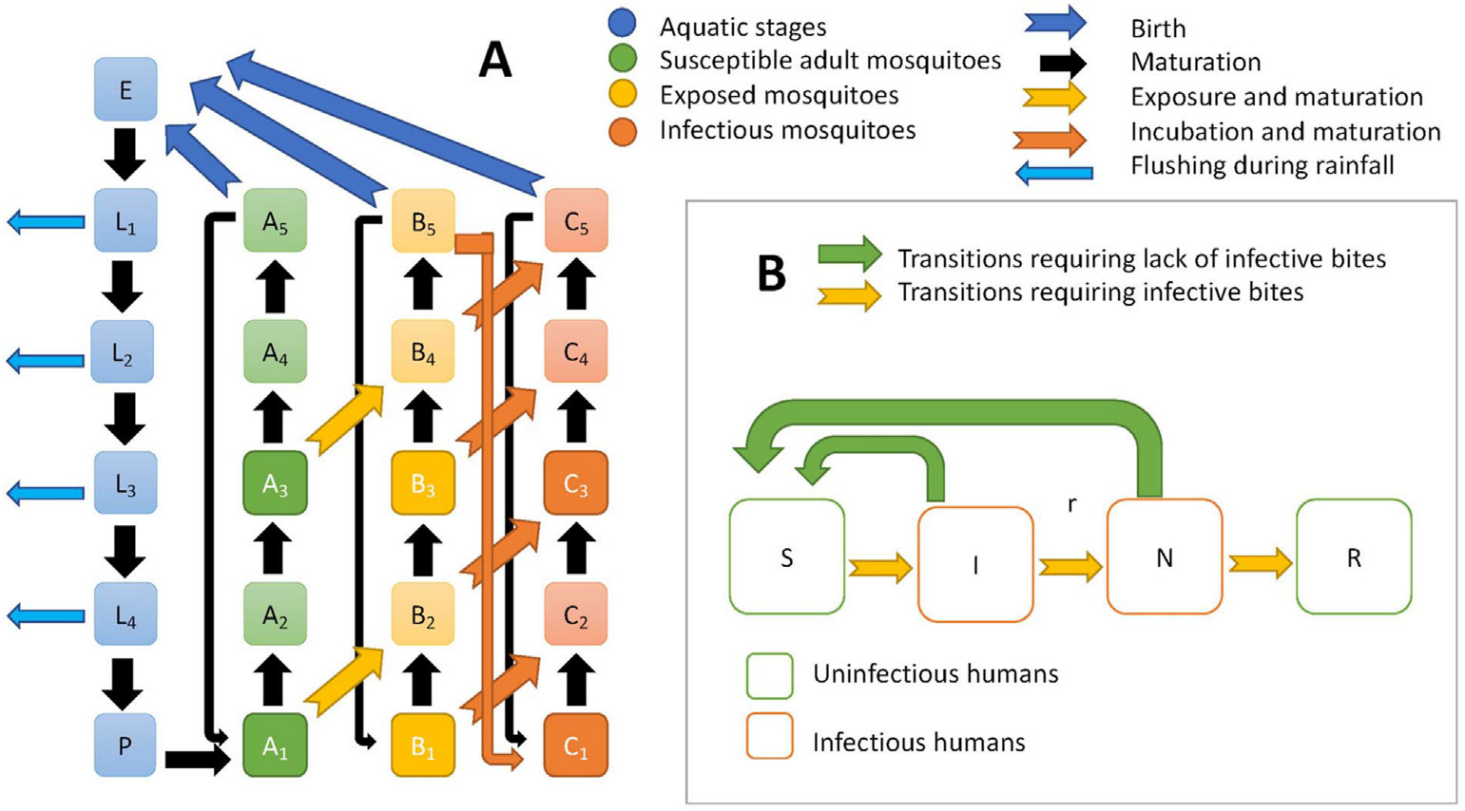
Diagram of the full model. **A** Vector life-cycle. **B** Disease transmission in humans. Light blue arrows represent flushing processes added to the model.

The 25 different equations describing the vector population and disease transmission system (diagrammed in Fig. 1) are taken from Ratti & Wallace (2020) with the addition of extra death terms due to flushing (indicated in boldface in equations in Supplementary Table S2). Model parameters representing hyperendemic and holoendemic transmission patterns in the absence of flushing are shown in Supplementary Table S1, along with temperature-dependent maturation parameters and rainfall associated larval habitat. Flushing rates for the four larval instars (*L*_1_, *L*_2_, *L*_3_, *L*_4_) are given by constants (*d*_1_, *d*_2_, *d*_3_, *d*_4_), varied in this study over a range of values. Flushing is implemented above a threshold value (*θ*). All remaining parameters are taken as in Supplementary Table S3. For the sake of comparison, we kept all the parameters the same as in Ratti & Wallace (2020) even though they can be re-calibrated.

Ratti & Wallace (2020) developed a model for malaria transmission driven by physical patterns of temperature and rainfall based on data from the Kakamega District in the Western Kenya highlands. This district includes towns with differing malaria prevalence patterns (Githeko et al., 2006).

The prior model accounts for all life stages of the insect vector as well as three epidemiological states of adult vectors: susceptible, infected during extrinsic incubation period, and infectious and able to transmit disease. Each of these three is separated into five sub-stages accounting for an approximate 5-day period between oviposition events. During this 5-day period, two days are allocated for questing and feeding behavior (Scott & Takken, 2012), two for resting and digesting after meals, and one for oviposition. All vectors are assumed to find a meal. Transition from one disease state to another is controlled by exposure to infective humans with a transmission term similar to that of the Ross-Macdonald model (Ross, 1911). Pre-adult stages assume no vertical transmission and are arranged as egg, four instars, and pupae. Human disease dynamics assumes four categories recognized in the literature: susceptible, infected, clinically immune, and long-term immune, with return to susceptible status possible relatively quickly for infected and less quickly for clinically immune individuals under conditions of low entomological infection rates.

Maturation rates for pre-adult stages depend on temperature and are parameterized according to laboratory studies, while overall abundance is controlled by the amount of available larval habitat (Ratti & Wallace, 2020). The model also includes larval density dependence on a stage-by-stage basis, with parameters for each stage estimated from instar ratios measured in field studies. Clearly, this is a simplification of the ecosystem of a natural puddle which includes crowding and predation, but adding further complexity makes the parameterization non-unique. For the purposes of that study and this one, it is only necessary to produce reasonable numbers of each stage, not duplicate the full dynamics in a particular habitat. Two parameters are mentioned to describe larval habitats in the system (*j*, average habitat and *c*, amplitude of habitat). These are incorporated into a truncated Fourier series modified from rainfall patterns for the region. Average habitat is the constant term in that series, while amplitude is multiplied by the sum of the oscillating terms. Varying these two parameters controls the average annual larval habitat and the rate to which it varies. For example, if the amplitude were zero, the model would represent permanent habitat available all year, and the prior model then predicts a holoendemic disease pattern if average habitat is high enough. If the amplitude varies a lot compared to the average, thus dropping to near zero for parts of the year, the model predicts a hyperendemic pattern if average habitat is high enough. The model does not account explicitly for different kinds of habitat (permanent, temporary, natural, man-made, etc.) but high amplitude variability implicitly means more temporary habitat.

By varying these two parameters, the prior model can produce hyper-, holo- and hypoendemic disease patterns. These patterns are characterized by an association between patterns of mosquito prevalence as measured by IRD (indoor residual density) and patterns of immunity usually characterized by age and immune status. The patterns produced specifically exclude many combinations of mosquito prevalence and immune status, giving at equilibrium only those associations observed for hyper-, holo- and hypoendemic disease patterns. These ranges are proof that the model is working correctly, as intermediate ranges are not observed (see the classification scheme in Supplementary Table S1). The prior model does not produce a mesoendemic pattern at equilibrium, and neither that study nor this one addresses intermediate patterns that may occur as disease is established in an area.

The model produces outputs for all quantities tracked. As the common classification scheme measures infection among younger individuals, and as long-term immunity tends to be acquired by older people, the ratio of infected individuals to susceptible plus infected plus clinically immune individuals is taken as an estimate of the percent of young people infected.

Benth et al. (2007) developed a weather simulation tool that extracts its parameters from an historical data stream, in this case rainfall. The simulation includes a deterministic aspect described by seasonal oscillations extracted as a Fourier series, a recursive aspect based on a one-day autocorrelation of the residual signal after removing the seasonal pattern, and the noise remaining after removing the seasonal pattern and the autocorrelation. A Fourier series is used to describe the time-varying standard deviation of the random noise. The parameters were extracted from 38 years of rainfall estimates for the Kakamega District (Sreekanth et al., 2020), shown in Supplementary Table S2. Rainfall estimates were obtained from the European Centre for Medium-Range Weather Forecasts (ECMWF) ERA-Interim reanalysis product (Berrisford et al., 2011). In the ERA-Interim system, regionally available observations are assimilated and merged with forecast model output, to produce estimates of surface, near-surface, and atmospheric properties at a 3- to 6-h interval with 80 × 80 km grid spacing (Dee et al., 2011). Daily mean precipitation (mm/day) from ERA-Interim were downloaded from the KNMI Climate Explorer (Oldenborgh, 2018) for the grid cell covering Kakamega, Kenya (0.27°N, 34.73°E) from 1 January 1979 to 28 February 2018. Let *P*(*n*) be the simulated data stream for daily rainfall, *F*(*n*) be the seasonal trend for that day, *r* the one-day lag autocorrelation, and *s*(*n*) be the seasonal trend for the standard deviation of remaining noise computed over a 7-day moving window. *R*(0, *s*(*n*)) is a random variable with mean 0 and standard deviation *s*(*n*). The formula for computing the simulated weather data values when only a single location is considered is given by:

(1) Q(n)=F(n)+r∗(P(n−1)−F(n−1))+R(0,s(n))

(simulated weather data values = seasonal trend + autocorrelation with previous day + noise with time-dependent standard deviation *s*(*n*) (Benth et al., 2007)). As rainfall cannot be negative, we set:

(2) P(n)=max(Q(n),0)

This model gives non-periodic daily rainfall that has the same seasonal trend and average daily rainfall as the data, with irregular variation that has the same autocorrelation as the data. The resulting model, which we will refer to as Monte Carlo Rainfall (MCRain), was used to select dates on which flushing events would occur, by selecting a threshold value for daily rainfall above which a flushing event is simulated with extra death rates (*d*_1_, *d*_2_, *d*_3_, *d*_4_), for each of the four instars. For numerical simulations we set *d*_3_ = *d*_4_ for large instars, and *d*_1_ = *d*_2_ = 1.5 ∗ *d*_3_ for small instars flushing at a slightly higher rate.

**Fig. 2:**
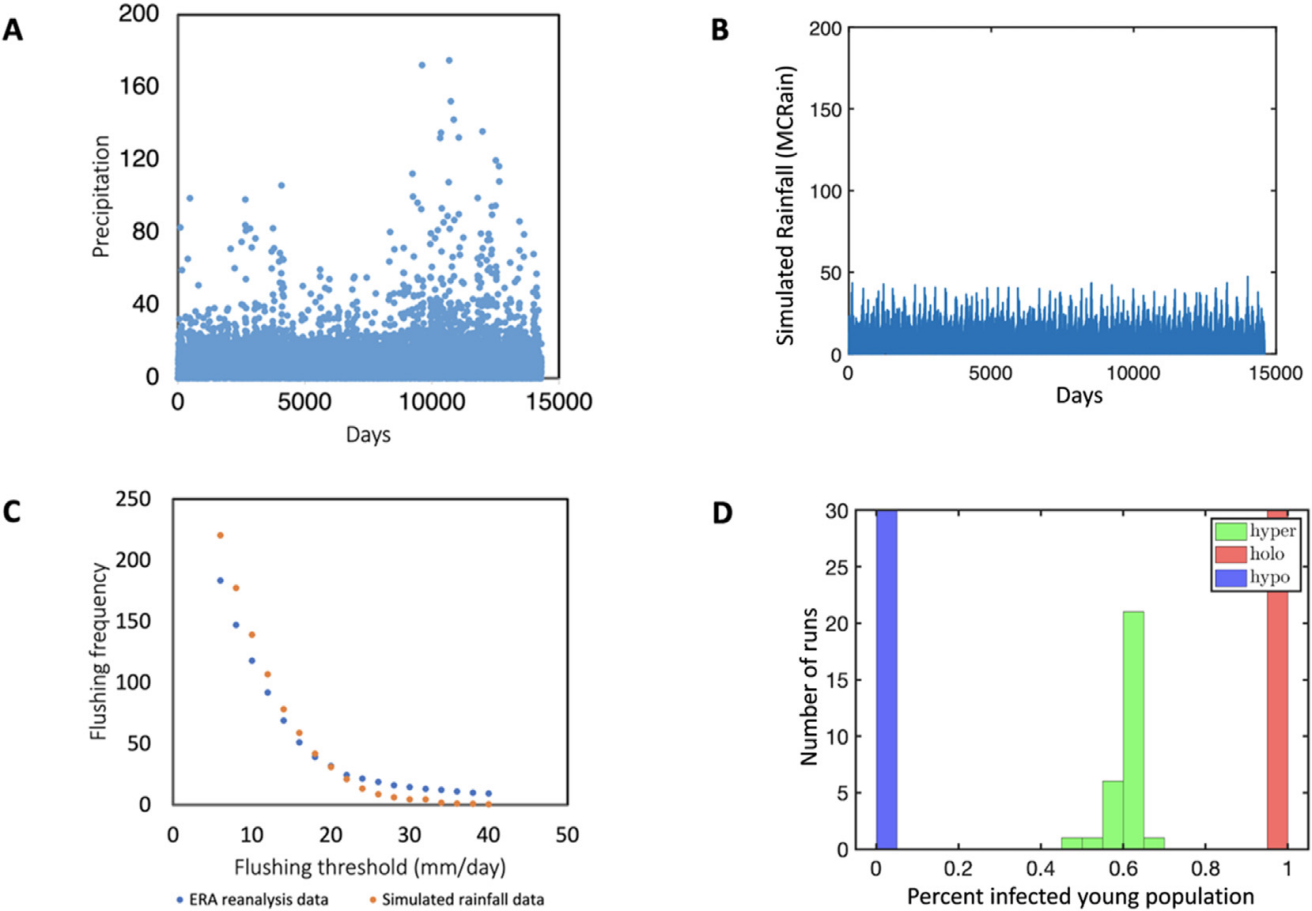
Monte Carlo rainfall performance. **A** Rainfall data. **B** Simulated rainfall. **C** Flushing threshold (mm/day) is shown on the horizontal axis and number of annual events, or flushing frequency, on the vertical. Note that the threshold range used in simulations was 20–40. **D** Robustness of the MCRain for different endemicity patterns.

One 10-year run of the Monte Carlo rain model (Fig. 2B) can be visually compared to ten years of the ERA dataset from which it was derived (2008–2017, Fig. 2A). Visually it is clear that the dataset has more extreme rainfall events than the model. The number of flushing events corresponding to each threshold for both model and data is shown in Fig. 2C.

To test the robustness under different runs of the rain simulation, two sets of parameters in each of the two scenarios were selected corresponding to hyper-, hypo- and holoendemic patterns. The Monte Carlo Rain model was run 30 times to give different patterns of rainfall, and the three scenarios were run to 40 years, using the 30 Monte Carlo Rain inputs. Percent infected young population $\left(\frac{I+C}{S+I+C}\right)\;$ was measured as an output variable for the last year.

Two scenarios from Ratti & Wallace (2020) were chosen that, in the absence of flushing, produce hyper- and holoendemic regions. Flushing was incorporated into each of these scenarios, and threshold and death rates varied to produce different frequencies of flushing events and different levels of flushing.

A 1.5:1 ratio of death rates was held constant to give a single death parameter, which is varied from *d*_3_ = 0 to 1 in increments of 0.1, independently of the threshold that controls frequency which was varied from 15 to 45 in increments of 2. The model was run to 40 years using *ode23 solver* in MATLAB, which produces a steady state disease pattern reflecting one of the WHO classifications.

## 3 Results

A mathematical model incorporating larval flushing was developed from an earlier model based on temperature and rainfall patterns obtained from reanalysis data for the Kakamega District in the western Kenya highlands. The prior model was able to produce hypo-, holo- and hyperendemic disease patterns depending on larval habitat availability corresponding to rainfall but varying in average amount and extremes of seasonal variation. The model developed in this study adds flushing events based on days of heavy rainfall. These are made to occur sporadically, but with appropriate seasonality and autocorrelation, using a Monte Carlo style simulation developed by Benth et al. (2007). The model used to initiate flushing events gives the correct mean daily rainfall and characteristic seasonal pattern of two rainy seasons, as shown by the reanalysis data in Fig. 2A and the simulated rainfall in Fig. 2B.

The resulting frequency of high rainfall events given by the model is similar to the frequency predicted by 30 years of reanalysis data, especially for flushing thresholds in an intermediate range, as illustrated in Fig. 2C. The model produces somewhat more days above a given threshold at low thresholds, and somewhat fewer days at high threshold. In the range explored in this study, between 15 mm/day and 30 mm/day, the model is in good agreement with the data. The model is run to 40 years to achieve a steady state. Although there is a random component to the Monte Carlo model, Fig. 2D shows that basic predictions of endemic classification are robust under repeated iterations.

**Fig. 3:**
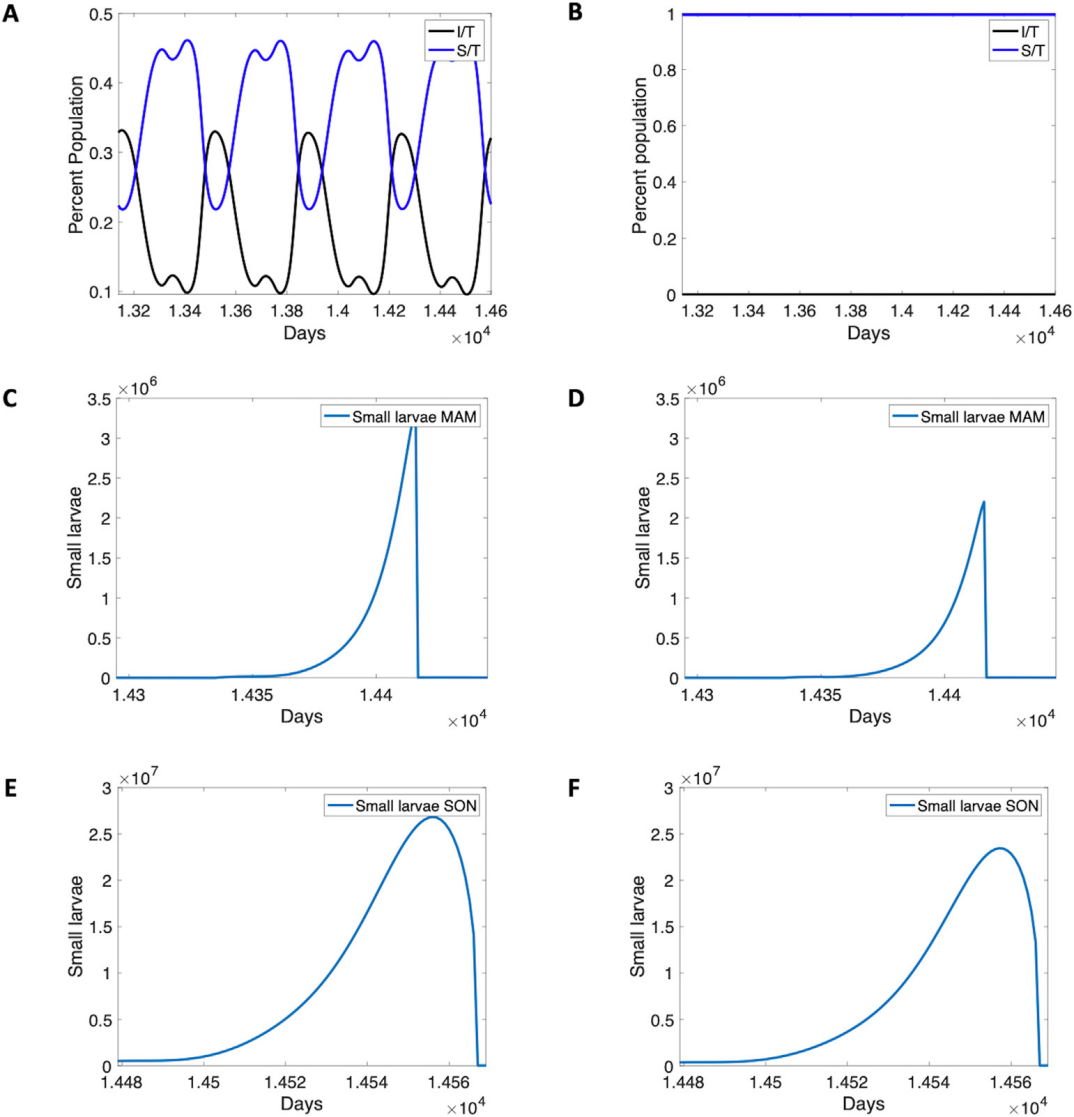
Hyperendemic to hypoendemic transition. Results are shown without flushing on the left, and with flushing on the right. The model is run with *S*_0_ = 19,149 and *R*_0_ = 1000, as well as many mosquitoes (see default initial conditions), for a 40-year time frame. **A** Typical hyperendemic seasonal pattern of disease in last five years. Blue indicates susceptible population. No flushing is included (and *j* = 1000*, c* = 3). **B** Model with flushing. With flushing included, nearly all are susceptible (blue line above 80%, *θ* = 25, *d*_3_ = *d*_4_ = 0.2, *d*_1_ = *d*_2_ = 0.3). Remaining population are remnants of immune classes, slowly declining (not shown). **C** and **E** Model without flushing. With no flushing, small larvae populations rise in the two rainy seasons approximated here by March-April-May (MAM) (**C**) and September-October-November (SON) (**E**) respectively. Year 40 is shown. **D** and **F** Model with flushing. With flushing included for the 40-year period, larval populations are substantially depressed in both seasons, with visible flushing events in the MAM season of Year 40, shown.

Flushing events can make the difference between hyperendemic and holoendemic disease patterns and hypoendemic disease, depending on the frequency of flushing events and the intensity of larvae removal. An example of this phenomenon is shown in Fig. 3. On the left in panels A, C and E is an example of hyperendemic transmission patterns. Fig. 2A shows the seasonal pattern of infection, while Fig. 3C and E shows the March-April-May (MAM) and September-October-November (SON) seasons of the last year of the run. On the right in panels B, D, F, flushing has been introduced; Fig. 3B shows the resulting disease-free steady state, and Fig. 3D and F shows the MAM and SON seasons. Flushing events are more frequent in the MAM season, although both show far fewer larvae present than without flushing. In this case, flushing over a 40-year period creates a hypoendemic disease pattern in which disease has died out with mosquitoes still present in the system.

**Fig. 4:**
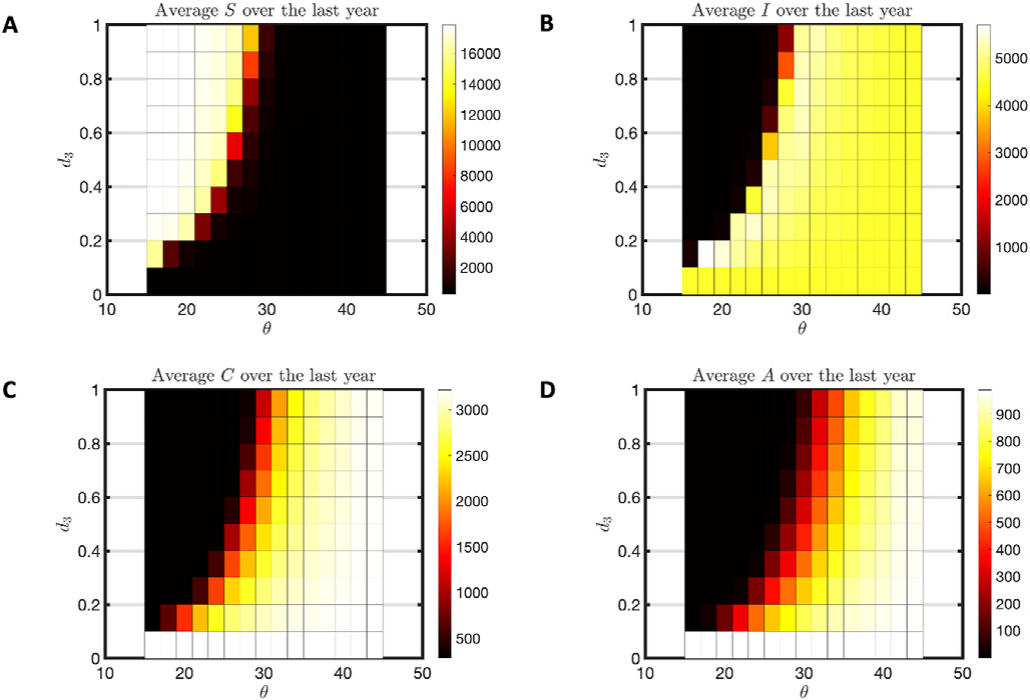
Hyperendemic to hypoendemic heatmap for humans. This heatmap is for a hyperendemic region (*j* = 1000 and *c* = 3). We varied *d*_3_ from 0 to 1 with a step size of 0.1 and used *d*_1_ = *d*_3_ ∗ 1.5. The symbol *θ* is used for threshold of flushing and it is varied from 15 to 45 with an increment of 2. For each combination of *d*_3_ and *θ*, the model was run for 40 years. **A** The average susceptible (S) population over the 40th year. **B** The average infected (I) population over the 40th year. **C** The average clinically immune (C) population over the 40th year. **D** The average of long-term immune (A) population over the 40th year.

**Fig. 5:**
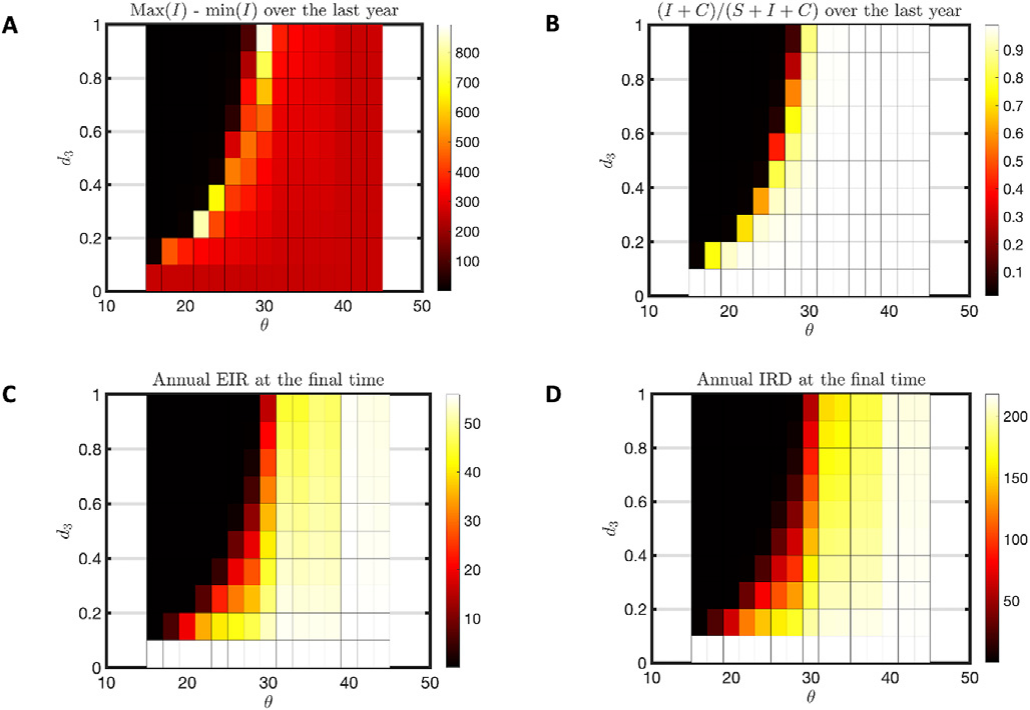
Hyperendemic to hypoendemic heatmap for seasonality in prevalence and insect abundance. This heatmap is for a hyperendemic region (*j* = 1000 and *c* = 3). We varied *d*_3_ from 0 to 1 with a step size of 0.1 and used *d*_1_ = *d*_3_ ∗ 1.5. The symbol *θ* is used for threshold of flushing and it is varied from 15 to 45 with an increment of 2. For each combination of *d*_3_ and *θ*, the model was run for 40 years. **A** The difference between maximum infected (max(I)) population and minimum infected (min(I)) population over the 40th year. **B** The percent infected young population (I + C/S + I + C) over the 40th year. **C** The annual EIR at the last day of simulation for each combination of *d*_3_ and *θ*. **D** The annual IRD at the last day of simulation.

Flushing induces hypoendemic disease patterns out of a hyperendemic habitat pattern, as seen across a range of flushing thresholds and intensities in Fig. 4. As the threshold rises (creating fewer flushing events) or the death rate due to flushing declines, one sees a hyperendemic pattern with few susceptible, but many infected, clinically immune and long-term immune people (Fig. 4A–D) as well as high seasonal variation in infection (Fig. 5A), high percent of infectious individuals (Fig. 5B), high EIR (Fig. 5C) and IRD (Fig. 5D). For low threshold and higher death rates one sees the absence of disease and a hypoendemic pattern. Comparing Fig. 5C and D, note the intermediate range where the EIR has dropped to zero, but the IRD is still non-zero. These are parameters for which mosquitoes are present but not disease, as shown in Fig. 3.

A thorough study of the model without flushing was done in prior work (Ratti & Wallace, 2020). The model was able to produce only hypo-, holo- and hyperendemic disease transmission patterns at steady state. No parameter choices produced anomalous combinations of disease transmission and insect abundance. However, the mesoendemic transmission pattern was noticeably absent. With the introduction of flushing, it has appeared as a category of steady state behavior at the boundary of the hyper to hypoendemic transition in Fig. 4. Mesoendemic transmission is characterized by a prevalence of 11–50%, fluctuating transmission pattern and EIR near 10 (Last et al., 2001). Note the relevant measures for *θ* = 28 and death (*d*_3_) = 0.1 in Fig. 5 for an example of this behavior.

**Fig. 6:**
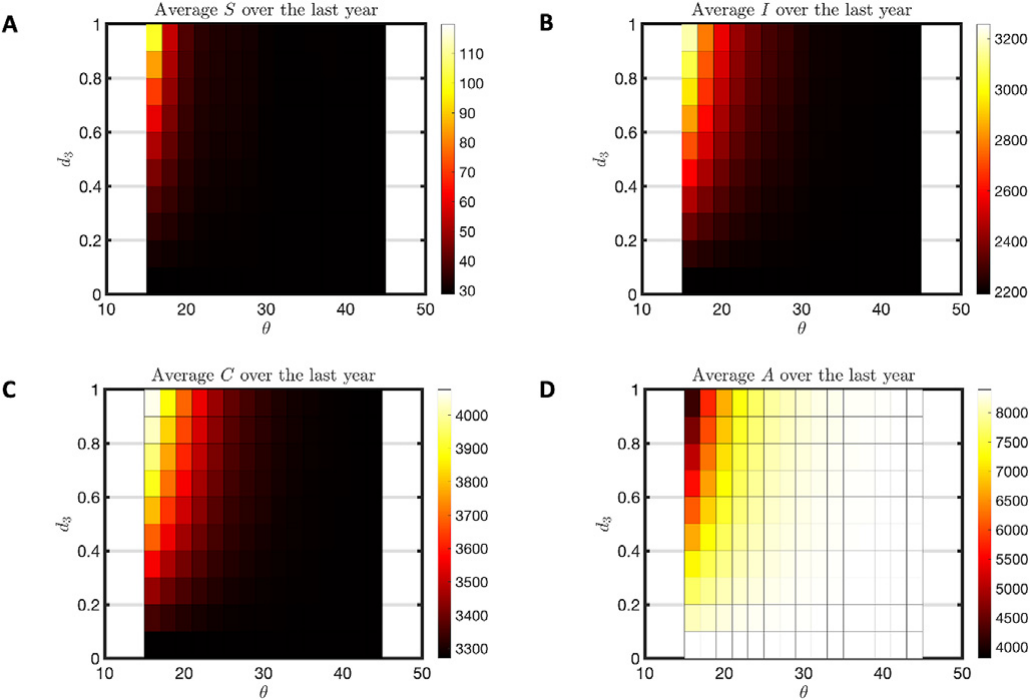
Holoendemic heatmap for humans. This heatmap is for a holoendemic region (*j* = 20,100 and *c* = 1). We varied d_3_ from 0 to 1 with a step size of 0.1 and used d_1_ = d_3_ ∗ 1.5. The symbol θ is used for threshold of flushing and it is varied from 15 to 45 with an increment of 2. For each combination of d_3_ and θ, the model was run for 40 years. **A** The average susceptible (S) population over the 40th year. **B** The average infected (I) population over the 40th year. **C** The average clinically immune (C) population over the 40th year. **D** The average of long-term immune (A) population over the 40th year.

**Fig. 7:**
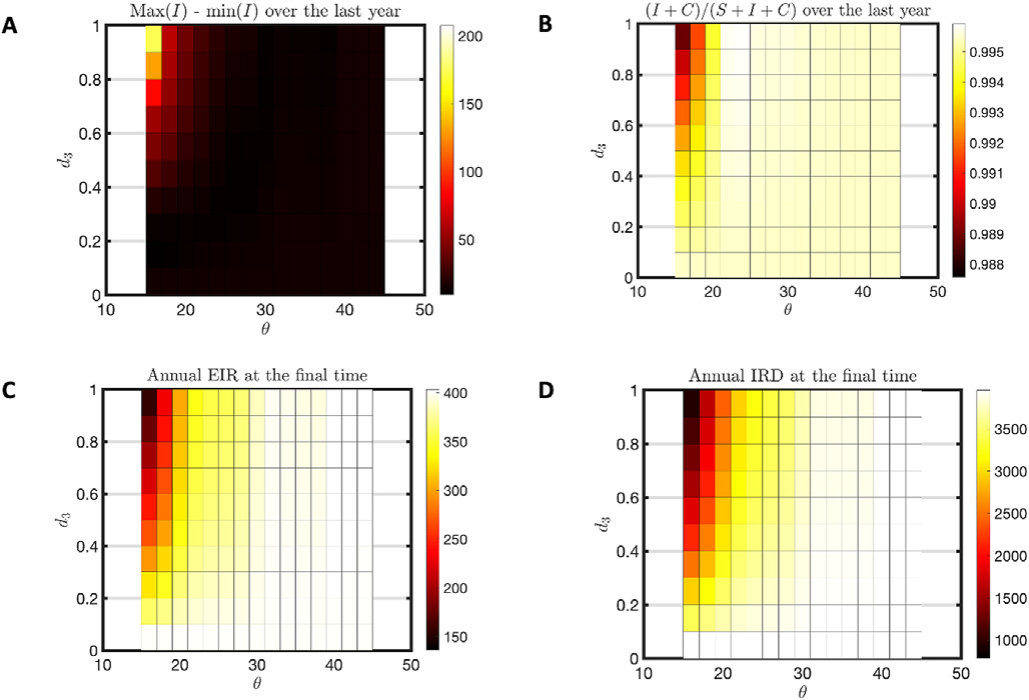
Holoendemic heatmap for seasonality in prevalence and insect abundance. This heatmap is for a holoendemic region (*j* = 20,100*, c* = 1). We varied *d*_3_ from 0 to 1 with a step size of 0.1 and used *d*_1_ = *d*_3_ ∗ 1.5. The symbol *θ* is used for threshold of flushing and it is varied from 15 to 45 with an increment of 2. For each combination of d_3_ and *θ*, the model was run for 40 years. **A** The difference between maximum infected (max(I)) population and minimum infected (min(I)) population over the 40th year. **B** The percent infected young population (I + C/S + I + C) over the 40th year. **C** The annual EIR at the last day of simulation for each combination of *d*_3_ and *θ*. **D** The annual IRD at the last day of simulation.

An early model on which this work is based was created to describe an endemic malaria transmission in the town of Emutete based on IRD data from colleagues at Kenya Medical Research Institute (Wallace et al., 2016). That early model corresponds to habitat parameters *j* = 201,000 and *c* = 1, with no flushing (high threshold, low death) in Fig. 6, producing a holoendemic disease pattern with larval habitat present year-round. In this situation, adding flushing does nothing to change the endemicity, as clearly illustrated in Fig. 6 and validated by temporal plots (not shown). Immunity remains high (Fig. 6C and D), few are susceptible (Fig. 6A) and there is little seasonal variation (Fig. 7A). Mosquitoes are abundantly present at all flushing levels (Fig. 7C and D).

**Fig. 8:**
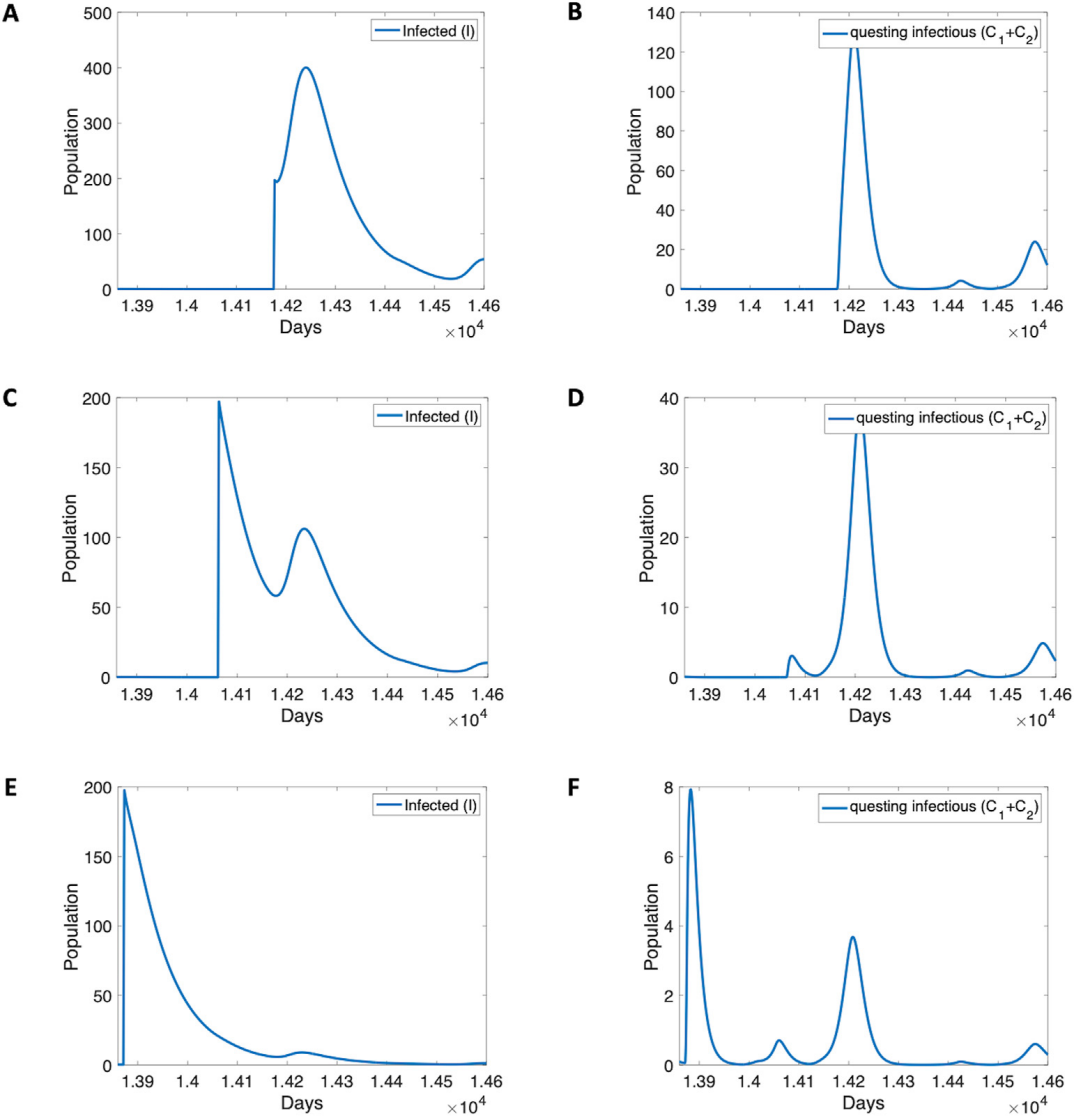
Outbreaks in flushing-induced hypoendemic regions. The model was run for 40 years with no initial infections (*j* = 1000, *c* = 3, *d*_3_ = *d*_4_ = 0.2, *d*_1_ = *d*_2_ = 0.6, *θ* = 30, *R*_0_ = 0). At various time points infectious humans (*I*) were introduced at 100 per day for 2 days. Model responses include outbreak (**A**), delayed outbreak (**C**) and no outbreak (**E**). A Infected humans after introduction on day 14,175, October 1st of year 39. **B** Infected questing mosquitoes after introduction on day 14,175, October 1st of year 39. **C** Infected humans after introduction on day 14,062, July 11th of year 39. **D** Infected questing mosquitoes after introduction on day 14,062, July 11th of year 39. **E** Infected humans after introduction on day 13,871, January 1st of year 39. **F** Infected questing mosquitoes after introduction on day 13,871, January 1st of year 39.

Hypoendemic regions with mosquitoes present are still prone to outbreaks if disease is introduced at certain timepoints, as shown in Fig. 8. Introducing 200 infected individuals on October 1st, during the SON rainy season, created an outbreak with a peak of over 500 infected individuals (Fig. 8A). When introduced on July 11th, between the rainy seasons, there was no initial outbreak but a delayed secondary outbreak of 150 or so individuals occur later as infected vectors peak (Fig. 8C and D). When infections are introduced on January 1st, during the dry season, no outbreak occurs at all (Fig. 8A).

## 4 Discussion

Malaria transmission patterns in the western Kenya highlands are distinguished by their heterogeneity (Wanjala et al., 2011; Imbahale et al., 2012). The effects of topography, land use, and weather patterns are usually cited as causes for this heterogeneity (Githeko et al., 2006; Atieli et al., 2011; Smith et al., 2013; Wanjala & Kweka, 2016). However, flushing of larvae from habitats is an acknowledged phenomenon that may distinguish otherwise similar habitat availability (Paaijmans et al., 2007; Dieng et al., 2012; Hardy et al., 2013; Mbouna et al., 2019). Larval habitat patterns that would create a hyperendemic prevalence without flushing can become hypoendemic with sufficiently extreme flushing, as shown in Fig. 4, Fig. 5. Holoendemic prevalence patterns, however, remain stable in the presence of high levels of flushing (Fig. 6, Fig. 7). In the model developed here, mesoendemic patterns do not emerge at steady state unless flushing is included, at the boundary between hyper- and hypoendemic patterns in Fig. 4, Fig. 5. Flushing can create a pattern in which the vector population dynamics do not necessarily run in parallel with the amount of habitat present and offer one mechanism by which the vector remains viable while disease is not present, as shown in Fig. 3. Such a pattern can produce outbreaks of disease depending on the time of year when it is introduced, as shown in Fig. 8. In short, flushing phenomena explain a variety of prevalence observations, including heterogeneity, patterns of outbreak, and associations with topographical properties.

### 4.1 Novelty of the model

The VECTRI model of Tompkins & Ermert (2013) incorporates flushing as an exponential function of rain rate but does not include immune states of humans. It uses parasite ratios and EIR as proxies for endemicity but does not distinguish between hyper- and holoendemic patterns. Many subsequent modeling efforts rely on this model, applying it to various locations. Habitat formation and disappearance has been approached using hydrology models (Bomblies et al., 2008).

Flushing in this model is portrayed as discrete events rather than a continuous removal based on rainfall. The model indicates that changes in rainfall parameters (mean and variability) can alter the endemicity pattern and, in particular, drive hyperendemic regions into the hypoendemic range. Such transitions are observed in the field as a result of control interventions (Tang, 2000).

The model presented here includes two immune states of humans, which allows it to cleanly separate different endemicity classifications, compared to the continuous array of EIR produced by the VECTRI model. A recent review of progress in malaria modeling does not indicate any studies that are able to reproduce the malaria endemicity classification patterns commonly used by epidemiologists (Eikenberry & Gumel, 2018).

### 4.2 Performance of the Monte Carlo rainfall simulation

The Monte Carlo rainfall simulation used to trigger flushing events worked reasonably well, especially in the range of 15–30 mm/day, as shown in Fig. 2C. At extremely low values it consistently over-predicts the number of events and at extremely high values it under-predicts the number of events. In the range of 15–30 mm/day one can see a transition from hyper-to hypoendemicity in Fig. 4. On average 14.4 days per year exceed a threshold of 30 mm/day in the ERA data stream, whereas only 4.3 days exceed this threshold in the simulated data. At the lower end, 51 days exceed a threshold of 16 mm/day in the ERA data stream, whereas 58.8 days exceed this threshold in the simulated data. Although no direct measurements exist of flushing in the wild, a few studies of weather patterns in the Kenya estimate days of high rainfall or note erosion events due to high rainfall. Rainfall that causes erosion would have the effect of flushing the much lighter larvae of mosquitoes as well.

As part of an agricultural study, Guto et al. (2012) recorded weather data in the Meru South district of Eastern Kenya. The authors described a pattern of two rainy seasons similar to those in the model used in prior work (Ratti et al., 2018; Ratti & Wallace, 2020). Guto et al. (2012) gave daily rainfall amounts for 2007 and 2008. For the short rains in October–December of 2007, they recorded 12 instances of rainfall of more than 25 mm/day and 5 instances of rainfall of more than 40 mm/day. For the long rainy season from March-July of 2008 the same study recorded 7 instances of rainfall more than 25 mm/day and 2 instances of rainfall of more than 40 mm/day. During the short rainy seasons of 2007 and 2008 the authors found 3 and 6 erosion events due to high rainfall. During the long rainy seasons of 2007 and 2008 they found 2 erosion events in each of those years.

The observations of Guto et al. (2012) echo those of a much earlier study of the western Kenya highlands (Oliver, 1956) which observed, in addition, that most of the rainfall events are not local showers, but regional. In addition, half of the annual rainfall was observed to occur on only 13% of rainy days (Oliver, 1956), with a few big storms accounting for most of the rainfall. As the study found approximately 22 rainy days in each of the short and long rainy seasons, it follows that most of the rainfall in each of those seasons occurred on 2–4 days. Based on these two studies one would expect larval flushing to occur at least 2–6 times during a rainy season. It is likely that flushing is more frequent than this as some runoff would be present on other days as well.

Although such data are rare, these studies indicate that the number of flushing events produced by the model is reasonable. In Fig. 4, Fig. 5, a critical change occurs around the threshold of 30, corresponding to 4.3 days of flushing, which aligns well with the observations of runoff and erosion. One could also ask if the death rates due to flushing are reasonable. The result of simulated death rates in Fig. 4, Fig. 5 indicate interesting transitions in the 20–80% range. A study by Dieng et al. (2012) used artificial flushing of larvae in containers with “heavy” rain producing from 10% loss in a small container to over 80% loss in a large container. The death rates used in the model are within the realm of possibility.

We noted that the simulated rainfall data are not quite as extreme or heterogeneous as the real data. The novel use of Benth et al.’s (2007) Monte Carlo simulation on rainfall data combined with the use of a threshold for flushing events led to several design decisions that could have been made differently. The method of Benth et al. (2007) requires knowing a standard deviation for the residual noise used in the model, which was calculated over a 7-day moving window for the entire ERA data stream and then fit with its own truncated Fourier series. Both the size of the moving window and the number of terms used in the Fourier series are decisions that could have been made differently. Because rainfall data have high daily variability (compared to temperature, for example), increasing the magnitude of terms in the Fourier series does not go quickly to zero, as high frequencies start to capture daily variability. Both the series used for the original data and the noise were cut-off when the period was around a month to capture the notion of “seasonal” variability. Using fewer terms would result in more of the variation being allocated to the random noise component of the model. When computing the standard deviation for the random noise in the model, one does not want to include the seasonal trend if possible, so a window of just a week was used. Using a larger window would increase the variation. There has not yet been a study that indicates how to optimize these decisions.

### 4.3 Occurrence of mesoendemic patterns

The presence of flushing introduces cases where a mesoendemic pattern (11–50% prevalence and EIR near 10) is seen at steady state (*θ* = 28 and *d*_3_ = 0.1 in Fig. 4, Fig. 5). This pattern is not seen at equilibrium without flushing. This result suggests that mesoendemic patterns need not be transitory patterns as hypoendemic regions become gradually hyperendemic after disease is introduced. Rather, it appears that they can be present after the patterns of disease and immunity are stabilized. We note, however, that they occur in Fig. 4, Fig. 5 at the boundary of parameter space between hypo- and hyperendemic categories. Under conditions of climate change, parameters driving flushing are likely to be changing. A slow change in parameters could allow a mesoendemic pattern to arise as a steady state after 30 years, but then disappear as the parameter moves into one of the other regions.

### 4.4 Limitations of the model

The model used in this study makes sense for a large population of insects, with results to be interpreted as the average result of flushing over a region. It is possible that flushing does not kill larvae, but instead moves them from one habitat to another where the runoff is collecting, and in this case “flushing” is interpreted as an average phenomenon over many habitats. It is possible that, for some habitats, high rainfall corresponds to a low death rate for larvae, while in others it corresponds to a high one. The model has only one death rate, which must average over all scenarios in the region. The model has a changing larval habitat but does not distinguish among habitats which shrink *versus* dry up completely, nor does it distinguish between habitats of high and low productivity. Nor is there a study in which these types of habitats are surveyed in terms of square meters. The more spatial resolution desired, the more these considerations matter.

### 4.5 Future work

It should be possible to compare the model output with observed data from the Western Kenya highlands; however, direct field measurements of flushing itself are not available. In addition, the introduction of larval death rates that correlate with synthetic rainfall-driven habitat does not in itself imply that flushing is the causal factor. To address these issues, it will be necessary to model the physical causes of flushing and connect these to factors in the landscape that have been measured.

For a given patch of habitat and quantity of precipitation, the probability of flushing is strongly affected by the magnitude of runoff, and thus indirectly by those factors that determine runoff, including soil properties, slope and other topographic variables, and vegetation/land cover. The runoff response to precipitation is often modeled using an empirical Curve Number (CN) approach (Ponce & Hawkins, 1996), originally developed in the United States but also used elsewhere (Deshmukh et al., 2013; Lal et al., 2017), or various process models such as the Soil and Water Assessment Tool (SWAT) (Todini, 1996; Arnold et al., 1998; Easton et al., 2008), ARNO (Todini, 1996), and variants of the Hydrologic Engineering Center rainfall/runoff model (HEC-HMS, 2021). The surface runoff Q can be estimated as follows:

$$Q=\frac{{\left(P-I_{a}\right)}^{2}}{P-I_{a}+S}$$

where *P* is the precipitation (and thus maximum potential surface runoff), *I*_*a*_ is the initial abstraction (including interception, evaporation, infiltration, and surface storage in depressions), and *S* is potential maximum retention, with all units in mm.

The most critical factors affecting runoff include antecedent soil moisture, topography, and land cover, all of which affect *I*_*a*_. When the soil is already at or near saturation, infiltration is negligible and additional precipitation will be overwhelmingly allocated to runoff (Olson et al., 2021). Steep slopes likewise reduce infiltration and surface storage, while vegetation cover increases interception and evapotranspiration and thus decreases runoff. Land cover change, including deforestation and the conversion of natural ecosystems to pasture or cropland, can have large impacts on time-integrated and peak discharge, increasing total runoff and the velocity and magnitude of the maximum runoff (Marsh, 1874; Olang & Fürst, 2011; Gessesse et al., 2015; Guzha et al., 2018). It has been observed that the time evolution of runoff can be modeled with fast and slow components (Jakeman & Hornberger, 1993), and a shift towards the fast component, and thus in peak discharge, may be particularly critical to flushing. These landscape factors explain why watersheds with similar climatology can have differing rates of runoff and flushing, driven by site-specific geomorphology and land cover. Making these connections explicit is the subject of a future study.

The Monte Carlo rain model, used to simulate daily rainfall amounts, assumes that the random noise component is normally distributed, and could be improved if the true distribution of the random noise component of the data were understood better. This improvement would bring the relationship of the flushing threshold and the number of flushing events into better alignment with the data.

In addition to flushing phenomena, the model presented here includes temporal changes in habitat, disease transmission, and two types of immunity. It is able to produce a full range of prevalence patterns and, more importantly, does not produce patterns other than those that have been observed. However, it does not yet include the effect of varying productivity among different types of habitat, which will also play a role in differentiating areas with the same weather pattern.

## 5 Conclusions

A model is presented that incorporates mosquito life stages, disease transmission, human immunity status, and sporadic flushing of aquatic larval habitat. The model produces known endemicity patterns at steady state and does not produce unobserved patterns. Simulations indicate that steady state mesoendemic disease patterns arise only when flushing phenomena are represented in the model, and also that frequent high rates of flushing can distinguish hyperendemic and hypoendemic patterns.

## Funding

Official funding for this study was not available.

## Ethical approval

Not applicable.

## CRediT author statement

Vardayani Ratti: conceptualization, methodology, software, visualization, writing - original draft preparation, writing - reviewing & editing, formal analysis. Dorothy Wallace: conceptualization, methodology, data curation, writing - original draft preparation, visualization, writing - reviewing & editing, formal analysis. Jonathan Chipman: conceptualization, methodology, writing - original draft preparation, writing - reviewing, investigation, validation. All authors read and approved the final manuscript.

## Declaration of competing interests

The authors declare that they have no known competing financial interests or personal relationships that could have appeared to influence the work reported in this paper.
