# Supplementary material for: Larval flushing alters malaria endemicity patterns in regions with similar habitat abundance
Source: Curr Res Parasitol Vector Borne Dis. 2022 Feb 8;2:100080. doi: 10.1016/j.crpvbd.2022.100080 (PMC9795365; doi:10.1016/j.crpvbd.2022.100080)
Supplement: Multimedia component 1 [file mmc1.pdf]

Supplementary Table S1. Properties of malaria prevalence classes

**Class**	**Prevalence**<br><br>**Spleen rate age 2-9**	**Transmission pattern Seasonality or other**	**EIR**<br><br>**Expected, observed**	**Immunity status Observed association**
Hypoendemic	0-10%	Little transmission (Baird et al., 2002)	Very low	
Mesoendemic	11-50% (Baird et al., 2002)<br><br>Proportional to EIR (Doolan et al., 2009)	Variable, fluctuates (Baird et al., 2002) with changes in one or many local conditions	Low, <10% (Doolan et al., 2009)<br><br>Bruce-Chwatt et al., 1973	
Hyperendemic	>50% (Baird et al., 2002)	Seasonal intense (Baird et al., 2002)<br><br>High in adults (Last et al., 2001)	periodic	Low in adults (Last et al., 2001)
Holoendemic	>75% (Baird et al., 2002)<br><br>$(24.2)\log_{10}(aEIR)+24.68$ (Baird et al., 2002)	Perennial intense (Baird et al., 2002) Low in adults (Last et al., 2001)	High, aEIR>10% (Doolan et al., 2009)	High in adults (Baird et al., 2002)

Supplementary Table S2 Governing equations

Eggs	$E' = b(A_5 + B_5 + C_5) - n_e E - q_e E$
1st larval stage	$L_1' = n_e E - n_1 L_1 - f_1 L_1 - \frac{q_1 L_1^2}{k} - \boldsymbol{d_1 L_1}$
2nd larval stage	$L_2' = n_1 L_1 - n_2 L_2 - f_2 L_2 - \frac{q_2 L_2^2}{k} - \boldsymbol{d_2 L_2}$
3rd larval stage	$L_3' = n_2 L_2 - n_3 L_3 - f_3 L_3 - \frac{q_3 L_3^2}{k} - \boldsymbol{d_3 L_3}$
4th larval stage	$L_4' = n_3 L_3 - n_4 L_4 - f_4 L_4 - \frac{q_4 L_4^2}{k} - \boldsymbol{d_4 L_4}$
Pupae	$P' = n_4 L_4 - n_p P - f_P P$
Susceptible biting female	$A_1' = \frac{n_P P}{2} + m A_5 - m A_1 - d_a A_1$
Susceptible resting female	$A_2' = m A_1 - \frac{mgI}{S + I + C + A} A_1 - \frac{mgC}{S + I + C + A} A_1 - m A_2 - d_a A_2$
Susceptible biting female	$A_3' = m A_2 - m A_3 - d_a A_3$
Susceptible resting female	$A_4' = m A_3 - \frac{mgI}{S + I + C + A} A_3 - \frac{mgC}{S + I + C + A} A_3 - m A_4 - d_a A_4$
Susceptible searching female	$A_5' = m A_4 - m A_5 - d_a A_5$
Infectious biting female	$B_1' = mF B_5 - m B_1 - d_a B_1$
Infectious resting female	$B_2' = \frac{mgI}{S + I + C + A} A_1 + \frac{mgC}{S + I + C + A} A_1 - mF B_3 - m B_2 - d_a B_2$
Infectious biting female	$B_3' = mF B_2 - m B_3 - d_a B_3$
Infectious resting female	$B_4' = \frac{mgI}{S + I + C + A} A_3 + \frac{mgC}{S + I + C + A} A_3 - mF B_3 - m B_4 - d_a B_4$
Infectious searching female	$B_5' = mF B_4 - m B_5 - d_a B_5$
Exposed biting female	$C_1' = m(1 - F) B_5 + m C_5 - m C_1 - d_a C_1$
Exposed resting female	$C_2' = m(1 - F) B_1 + m C_1 - m C_2 - d_a C_2$
Exposed biting female	$C_3' = m(1 - F) B_2 + m C_2 - m C_3 - d_a C_3$
Exposed resting female	$C_4' = m(1 - F) B_3 + m C_3 - m C_4 - d_a C_4$
Exposed searching female	$C_5' = m(1 - F) B_4 + m C_4 - m C_5 - d_a C_5$

Susceptible humans	$$S' = b_s - \frac{a(C_1 + C_3)}{S + I + C + A} S + \frac{\alpha}{\alpha + G} p_1 C + \frac{\alpha}{\alpha + G} p_2 I - d_h S$$
Infected humans	$$I' = \frac{a(C_1 + C_3)}{S + I + C + A} S - \frac{G}{\alpha_3 + G} p_3 I - \frac{\alpha_2}{\alpha_2 + G} p_2 I - d_i I$$
Clinically immune humans	$$C' = \frac{G}{\alpha_3 + G} p_3 I - \frac{\alpha_1}{\alpha_1 + G} p_1 C - \frac{G}{\alpha_4 + G} p_4 C - d_h C$$
Humans with parasite suppressing immunity	$$A' = \frac{G}{\alpha_4 + G} p_4 C - d_h A$$

Supplementary Table S3 Parameter values

Parameter	Description and dimensions	Value	Source
a	Probability of disease transmission from mosquitoes to humans (h/(mt))	(0.012 + 0.086)=2 = 0.049	Chitnis et al. (2008)
b	Egg laying rate	93.6	Hogg & Hurd (1997)
g	probability of acquiring infection when a susceptible mosquito feeds on infected human (dimensionless)	0.48	Chitnis et al. (2008)
d_a	Death rate of adult Mosquitoes (1/time)	0.167	Ratti et al. (2018)
d_h	Death rate of susceptible and partially immune humans(1/time)	0.000085	estimated
d_i	Death rate of infected Humans (1/time)	0.000255	Ngwa & Shu (2000)
$1 - F$	(length of the time for which)$^{-1}$=EIP^{-1}	1-1/12 =1- 0.0833	Kenya Birth Rate (2015)
b_s	Immigration rate of humans (h/t)	1.48	Ratti et al. (2018)
m	Probability of moving to the next day	1	Ratti et al. (2018)
j	Average habitat	135000 (baseline)	Ratti et al. (2018)
c	Amplitude habitat	1 (baseline)	Ratti et al. (2018)
p_1	Loss of clinical immunity	0.000575	Dobaño & Moncunill (2018); Achtman et al. (2005)
p_2	Loss of parasite suppressing immunity	0.0126	Bretscher et al. (2015)

p_3	Gain of clinical immunity	0.000821	Doolan et al. (2009)
p_4	Gain of parasite suppressing immunity	0.000410	Langhorne et al. (2008); Rogier et al. (1999); Tierlinck et al. (2011)
q_e	Density dependent parameters: eggs	0.0542	Wallace et al. (2016)
q_1	First instar L_1	0.000257	Wallace et al. (2016)
q_2	Second instar L_2	0.0008	Wallace et al. (2016)
q_3	Third instar L_3	0.0128	Wallace et al. (2016)
q_4	Fourth instar L_4	0.0229	Wallace et al. (2016)
$\alpha_{1,2,4}$	Immunity gain/loss	0.00274	Beier et al. (1999)
α_3	Gain of clinical immunity	0.0274	Doolan et al. (2009)
S_0	Initial susceptible population	19149	estimated
I_0	Initial infected population	0	estimated
C_0	Initial clinically immune population	1000	estimated
A_0	Initial population with parasite suppressing immunity	0	estimated
$A_{i0}, i = 1,2,\dots5$	Initial A_i population	1289400:7821495022=100=5	Ratti et al. (2018)
B_i	Initial infected mosquito population	0	estimated
C_i	Initial infectious mosquito population	0	estimated
E_0	Initial egg population	3954200.8835438923	Wallace et al. (2016)
L_{10}	Initial L_1 population	2310300.5386356956	Ratti et al. (2018)
L_{20}	Initial L_2 population	2007000.7121853685	Ratti et al.

			(2018)
L_{30}	Initial L_3 population	594100.3681122219	Ratti et al. (2018)
L_{40}	Initial L_4 population	215910.8303970495	Ratti et al. (2018)
P_0	Initial pupae population	103780.6956163843	Ratti et al. (2018)
